# Targeting Immunometabolism Mediated by CD73 Pathway in *EGFR*-Mutated Non-small Cell Lung Cancer: A New Hope for Overcoming Immune Resistance

**DOI:** 10.3389/fimmu.2020.01479

**Published:** 2020-07-14

**Authors:** Anna Passarelli, Michele Aieta, Alessandro Sgambato, Cesare Gridelli

**Affiliations:** ^1^Unit of Medical Oncology, Department of Onco-Hematology, Centro di Riferimento Oncologico della Basilicata (IRCCS-CROB), Rionero in Vulture, Italy; ^2^Laboratory of Pre-clinical and Translational Research, Centro di Riferimento Oncologico della Basilicata (IRCCS-CROB), Rionero in Vulture, Italy; ^3^Division of Medical Oncology, “S.G. Moscati” Hospital, Avellino, Italy

**Keywords:** non-small cell lung cancer, epidermal growth factor receptor, immune metabolism, adenosine, CD73, immunotherapy, immune resistance

## Abstract

Despite the relevant antitumor efficacy of immunotherapy in advanced non-small cell lung cancer (NSCLC), the results in patients whose cancer harbors activating epidermal growth factor receptor (*EGFR*) mutations are disappointing. The biological mechanisms underlying immune escape and both unresponsiveness and resistance to immunotherapy in *EGFR*-mutant NSCLC patients have been partially investigated. To this regard, lung cancer immune escape largely involves high amounts of adenosine within the tumor milieu with broad immunosuppressive effects. Indeed, besides immune checkpoint receptors and their ligands, other mechanisms inducing immunosuppression and including adenosine produced by ecto-nucleotidases CD39 and CD73 contribute to lung tumorigenesis and progression. Here, we review the clinical results of immune checkpoint inhibitors in *EGFR*-mutant NSCLC, focusing on the dynamic immune composition of *EGFR*-mutant tumor microenvironment. The adenosine pathway-mediated dysregulation of energy metabolism in tumor microenvironment is suggested as a potential mechanism involved in the immune escape process. Finally, we report the strong rationale for planning strategies of combination therapy with immune checkpoints blockade and adenosine signaling inhibition to overcome immune escape and immunotherapy resistance in *EGFR*-mutated NSCLC.

## Introduction

Primary lung cancer is the most common malignant tumor and the main cause of cancer-related death in the world (1). Non-small cell lung cancer (NSCLC) accounts for 80–90% of lung cancers, while small cell lung cancer (SCLC) has decreased in terms of frequency over the past decades (2, 3). The World Health Organization (WHO) calculates that lung cancers cause 1.5 million deaths per years and about 70% of them are due to smoking. However, recent advances in the field of anti-cancer therapies and mutations in oncogenic drivers (4), such as Epidermal Growth Factor Receptor (*EGFR*) mutations and Anaplastic Lymphoma Kinase (ALK) gene translocation, have improved the outlook in terms of both progression-free survival (PFS) and overall survival (OS), showing a promising future for advanced NSCLC therapy (5).

Tyrosine kinase inhibitors (TKIs) in oncogene-driven tumors and immunotherapy are the two major evolving strategies in the treatment of NSCLC (6, 7). To date, *EGFR*-TKIs are recommended by clinical guidelines as optimal first-line strategy in *EGFR*-mutated NSCLC (8). Despite initial responsiveness to *EGFR*-TKIs, acquired resistance within 9–18 months is almost inevitable (9). Therefore, the onset of acquired resistance to *EGFR*-TKIs has raised hopes of a role for immune checkpoint inhibitors (ICIs) characterized instead by durable response (10).

The use of immune checkpoint inhibitors (ICIs) has revolutionized the management of patients with non-oncogene addicted NSCLC in both first- and second-line settings, showing an unexpected long-term effectiveness and a good toxicity profile (11); on the other hand, to date the clinical outcomes of ICIs in oncogene-addicted NSCLC are disappointing.

To this regard, recent clinical studies have described limited efficacy of ICIs, targeting mainly cytotoxic T-lymphocyte antigen-4 (CTLA-4), programmed-cell death-1 (PD-1) or its ligand PD-L1, in NSCLC harboring *EGFR* mutations and TKIs naive (12). In addition, a recent work has suggested that the different molecular features of *EGFR* mutations in NSCLC may lead to a different responsiveness and outcomes to ICIs (13). It is noteworthy that *EGFR*-TKIs could modulate immune responsiveness to cancer by shaping the tumor microenvironment (TME) and enhancing ICIs benefit (14). Therefore, several clinical trials evaluating both efficacy and safety of immunotherapy combined with targeted therapy in patients *EGFR*-mutant NSCLC are currently ongoing (15, 16).

To date, several evidence have suggested that *EGFR*-mutated cancer cells represent a crucial hallmark of immunosuppression (17), actively establishing an immunosuppressive milieu and negatively influencing the quality of T-cell immune response.

Interestingly the immunosuppressive effects of *EGFR* mutations could also result in an immune metabolic dysfunction, in which an emerging role seems to be played by CD39/CD73 ectonucleotidases, catalyzing both over-production and release of extracellular adenosine (ADO), known as a powerful immunosuppressive nucleoside (18). In specific, the immune metabolic reprograming mediated by adenosine signaling in TME is reported as a further hallmark of *EGFR*-mutated NSCLC, with the precise aim to evade the immune surveillance and induce innate immune resistance to ICIs (19).

In this review, we discuss the role and features of TME in NSCLC harboring *EGFR*-mutation focusing on the involvement of immunometabolism mechanisms mediated by CD39/CD73—adenosine signaling. The potential application of targeting this pathway in the therapeutic strategy for overcoming the immunotherapy resistance is also evaluated.

## Immunotherapy in the Management of *EGFR*-Mutated Lung Cancer

The relevant success of the immunotherapy associated with favorable safety profile in advanced lung cancer treatment (20–23) suggests that, similarly to other cancer types, escape, or immune evasion processes concur to lung cancer pathogenesis and progression as well (24, 25). Indeed, monoclonal antibodies (mAb)s targeting PD-1, PD-L1, and CTLA-4 immune checkpoints leading to increased anti-tumor response due to increased T-cell activity and proliferation, have received regulatory approval across a wide range of tumors, including NSCLC (26). Specifically, patients with PD-L1 tumor proportion score (TPS) ≥50% are typically offered monotherapy with the anti-PD-1 mAb (Pembrolizumab®) (27). For patients with PD-L1 expression <50%, the combination of a platinum-doublet chemotherapy and Pembrolizumab has been recently approved (28). In addition, the combination of carboplatin plus paclitaxel with anti-angiogenic drug (Bevacizumab®) and anti-PD-L1 mAb (Atezolizumab®) represents an alternative treatment for patients with non-squamous NSCLC, which just received EMA and FDA approval (FDA approval excludes patients with *EGFR* or *ALK* genomic tumor aberrations) (29). In the second-line setting, rather than single-agent chemotherapy, Pembrolizumab has been approved for tumors that express PD-L1 (30), while Nivolumab and Atezolizumab represent a standard option regardless of tumor PD-L1 expression (31–33).

Despite promising advances in immunotherapy, the role of ICIs in oncogene-addicted NSCLC remains unclear and conflicting. The majority of data come from subgroup analyses with low number of patients, therefore the use of ICIs, when permitted by regulatory agencies, should only be considered when other available therapies, including standard *EGFR*-TKIs, fail (34).

Unfortunately, after failure of first-line TKIs, patients with *EGFR* mutations have limited treatment options. Two meta-analysis covering several clinical trials observed relatively poor efficacy and low response rates to PD-1/PD-L1 inhibitors vs. standard second-line chemotherapy among patients with pre-treated *EGFR*-mutant lung cancer (35, 36). Based on molecular status, the OS improvement was confirmed for *EGFR* wild-type lung cancers (OS hazard ratio (HR): 0.67; *p* < 0.001], but not in those *EGFR* mutated (OS HR: 1.11; *p* = 0.54), although no clear conclusions can be drawn due to the limited number of patients as part of subgroup analyses.

Of most interest, Lisberg et al. reported the role of immunotherapy with Pembrolizumab as first-line for *EGFR*-mutant NSCLC patients with PD-L1 expression of at least 1%, confirming once again the failure of immunotherapy alone for *EGFR* mutant patients (12). Although this trial evaluated Pembrolizumab in only 10 *EGFR*-mutant, TKI naïve patients, the lack of efficacy in terms of objective response reported was striking, especially since 70% of these patients had PD-L1 expression ≥50% (12, 37). About this, an interesting retrospective analysis showed that among *EGFR*-mutated NSCLC patients with high PD-L1 expression (TPS ≥50%) the efficacy of PD-1 inhibitors tended to be lower as compared to *EGFR* wild-type patients (38). These disappointing results could be related, at least partly, to the genomic landscape of *EGFR*-mutant NSCLC (39). Indeed, this type of cancer shapes a typical “uninflamed” TME characterized by a lack of T-cell infiltration, a shrinking proportion of PD-L1^+^/CD8^+^ tumor infiltrating lymphocytes (TILs), an immune metabolic reprogramming process and a lower mutation burden (17). Therefore, the limited benefit of immunotherapy in *EGFR*-mutant patients has led to alternative approaches or rather combination strategies targeting several pathways. These include ICIs plus an anti-angiogenic therapy or *EGFR*-TKIs (Table 1), as well as ICIs with chemotherapy to increase immunogenicity of *EGFR*-mutant tumors and responsiveness to ICIs (40).

**Table 1:** Clinical trials of immunotherapy combined with EGFR-TKi in EGFR-mutated NSCLC.

	**Drugs Combination**				**Outcome**	**Safety**	
**Clinical trial**	**TKi**	**ICI**	**Condition or disease**	**Phase**	**ORR (%)**	**AEs Grade 3-4**	**Trial Status**
NCT02088112	Gefitinib	Durvalumab	EGFRm NSCLC, TKi naive	I	63%	58%	Active, not recruiting
NCT02013219	Erlotinib	Atezolizumab	EGFRm NSCLC, TKi naïve or previously treated (without TKi)	I	75%	50%	Active, not recruiting
-	Erlotinib	Nivolumab	EGFRm NSCLC, TKi naïve or treated	I/II	15%	25%	Completed
NCT02143466TATTON	Osimertinib	Durvalumab	EGFRm NSCLC, TKi naïve or treated	I	43%	48% (ILD-transaminitis)	Active, not recruiting
NCT02454933CAURAL	Osimertinib	Durvalumab	EGFR-T790M NSCLC previously treated with TKi	III	64%	8%	Active, not recruiting
NCT02039674	Erlotinib/Gefitinib	Pembrolizumab	EGFRm NSCLC	I/II	-	-	Active, not recruiting

*Source from ClinicalTrials.gov*.

*TKi, tyrosin-kinase inhibitor; ICI, immune checkpoint inhibitor; ORR, Overall Response Rate; AEs, Adverse Events; EGFR, epidermal growth factor receptor; NSCLC, Non-Small-Cell Lung Cancer; N/A, not available; ILD, Interstitial Lung Disease*.

Importantly, the IMpower150 trial reported that in *EGFR*-mutant NSCLC patients the addition of Atezolizumab to Bevacizumab plus carboplatin plus paclitaxel (ABCP) provided significant clinical benefit (41). In specific, in a subset analysis of *EGFR*-mutant patients treated after TKI failure, median OS was not estimable for patients treated with the addition of ABCP vs. 17.5 months for patients treated with Bevacizumab, carboplatin plus paclitaxel (BCP) (HR 0.31; 95% CI 0.11–0.83); median PFS was 10.3 months with ABCP vs. 6.1 months with BCP, associated with a similar and good safety profile (39). However, the subgroup analysis and the very low patients number (overall 114 patients) with activating *EGFR* mutation or *EML4-ALK* rearrangement status are important limitations.

Furthermore, preclinical studies reported an immune modulatory effect of *EGFR* signaling by regulating expression of MHC I/II and PD-L1 on tumor cells and the activity of T-cells. This suggests a potential synergistic effect for the use of immunotherapy in combination with *EGFR*-TKIs (42), according to the recent evidence of long-lasting antitumor responses of *BRAF/MEK* inhibitors with immunotherapy in the treatment of *BRAF*-mutated metastatic melanoma (43). However, this promising combination strategy remains controversial due to the significant toxicity observed in several clinical trials following administration of anti-PD-(L)1 mAbs in combination with *EGFR*-TKIs (44, 45) (see Table 1).

In conclusion, all these studies suggest that further and prospective clinical trials with different and less toxic drugs are required to better define if there is a role for ICIs strategy in the treatment of oncogene-addicted NSCLC.

## The Immune Microenvironment in EGFR-Mutant NSCLC

The TME composition plays a considerable role in tumor growth and progression (24). Surprisingly, the TME is able to act as either obstacle or facilitator of cancer proliferation and progression by affecting several biological mechanisms. During tumorigenesis, both immune and *EGFR*-mutant tumor cells are subjected to the immunoediting process consisting of dynamic and interconnected phases, including elimination, equilibrium, and finally immune evasion. Therefore, this complex interplay is essential to define appropriate strategies to target TME as part of the anti-cancer therapy.

**Figure 1:**
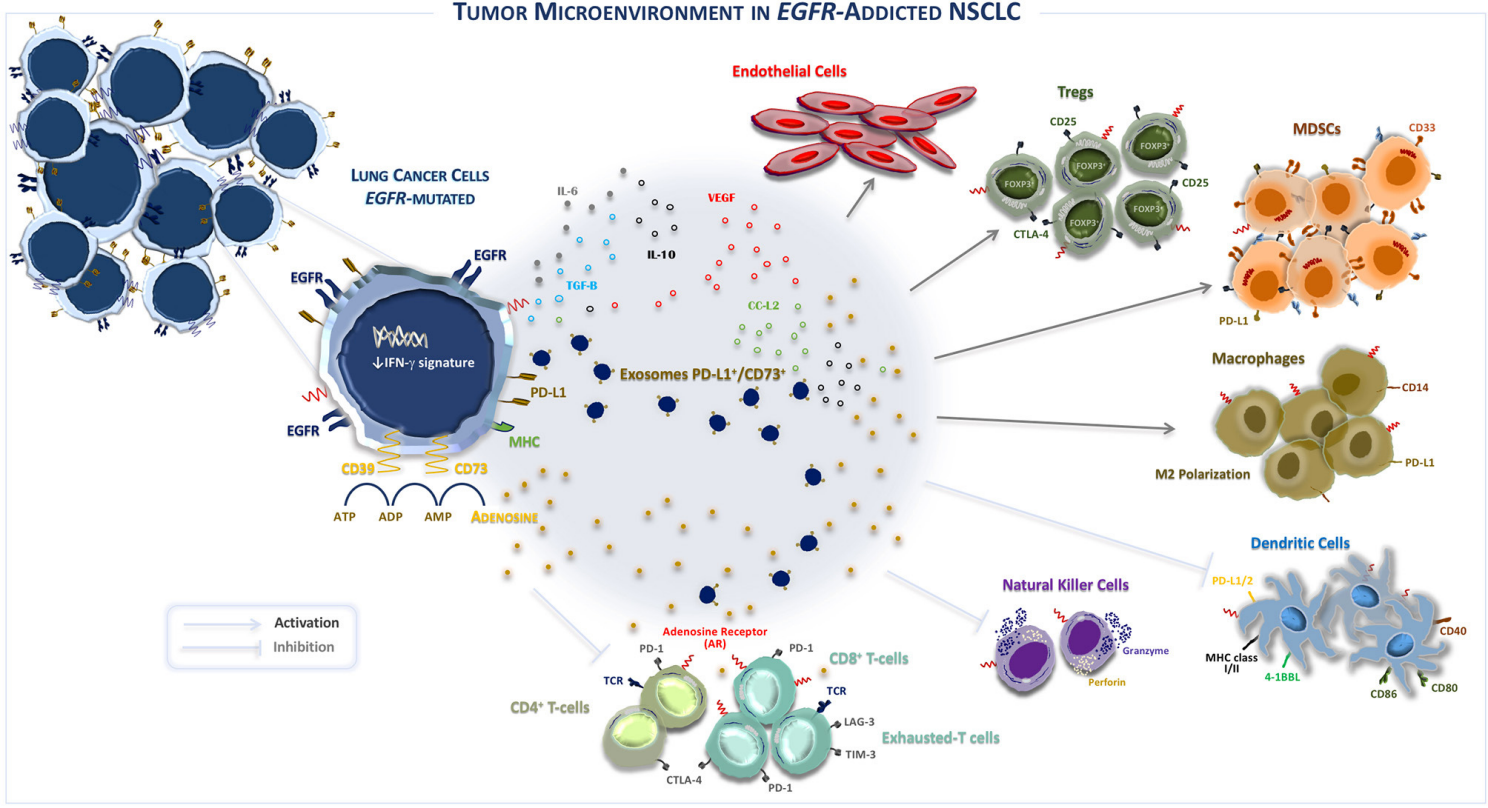
The tumor microenvironment in EGFR-addicted NSCLC. *EGFR*-mutated NSCLC is typically characterized by an “uninflamed” tumor microenvironment, immunological tolerance, and weak immunogenicity. Recently, it was suggested that the over-expression of CD39/CD73—adenosine signaling also induces an immunosuppressive TME. Indeed, CD39/CD73 ectonucleotidases are widely expressed by lung cancer cells and induce the high extracellular production and release of immunosuppressive adenosine that shapes the activity of innate and adaptive immune system cells and endothelial cells in TME. Specifically, the activation of CD39 induces the de-phosphorylation of ATP to ADP and, subsequently, to AMP, while CD73 catalyzes the hydrolysis of AMP into adenosine and phosphate. By binding the A2A adenosine receptor A2AR, the most common receptor subtype expressed by both adaptive and innate immunity, extracellular adenosine induces inhibitory signals in TME that restrain the activity of immune system cells, promoting growth and survival of *EGFR*-mutated lung cancer cells. In addition, exosomes derived by tumor cells also contribute to modulate immunosuppression by influencing PD-L1^+^/CD73^+^ expression and extracellular adenosine release. EGFR, Epidermal growth factor receptor; Tregs, T regulatory T-cells; MHC, Major histocompatibility complex; PD-1, Programmed cell death protein; CTLA-4, Cytotoxic T lymphocyte antigen-4; MDSCs, Myeloid-derived suppressor cells; TCR, T-cell receptor; VEGF, Vascular endothelial growth factor; IL, Interleukin; M2, Macrophages 2; ATP, Adenosine triphosphate; ADP, Adenosine diphosphate; AMP, Adenosine monophosphate; CCL2, C-C motif chemokine ligand 2; FoxP3, forkhead box P3.

To this regard, recently it has been reported that the TME of *EGFR*-mutated NSCLC concurs to create an immunosuppressive milieu, as represented in Figure 1 (46). In fact, immunosuppressive effects of *EGFR* mutations shape both composition and function of TME by interfering with several intracellular pathways and modulating immune accessory cells such as tumor-infiltrating lymphocytes (TILs), natural killer (NK) cells, T-regulatory cells (Tregs), myeloid-derived suppressor cells (MDSCs), tumor-associated macrophages (TAMs), involved in the increased release of immunoregulatory soluble factors such as cytokines and exosomes, as summarized in Table 2 (47, 48).

**Table 2:** The major hallmarks and mechanisms of Tumor Immune Escape in the TME of EGFR-mutated non-small cell lung cancer.

**Mechanism of action**	**Population(s)/pathway(s)**	**Effect(s) on TME**
Tumor-infiltrating lymphocytes (TIL)s	CD3^+^ lymphocytes CD8^+^ T-cells CD4^+^ T-cells	Inefficacy tumor-cytotoxicity of T-cells
Over-expression of inhibitory immune checkpoints	↑ CTLA-4, PD-1, LAG3, TIM3, VISTA, TIGIT	Inhibition of T-cell function/T-cell anergy
Over-expression of immune checkpoint ligands	PD-L1 PD-L2	Activation of inhibitory immune checkpoints
Defective recognition of lung cancer cells	Downregulation, mutation or loss of MHC class I Lung cancer antigens Defective antigen presentation	↓ Initiation of the antigen-specific immune response, antigen processing and presentation, and the cross-priming processesInefficacy activation of lung cancer infiltrating T-cells
Up-regulation of immune suppressive cells	MDSCs CD4^+^CD25^+^FoxP3^+^ Tregs Macrophage infiltration (M2-TAM)	Inhibition of T-cell function Direct pro-tumorigenic effect (VEGF, TGF-β)
Release of pro-tumorigenic and pro-angiogenic factors by TME	TGF-β VEGF, IL-10, IL-6, MMP CCL-2 iNOS	Induction of both recruitment and accumulation of immunosuppressive cells Tumor angiogenesis and stroma remodeling Migration of MDSCs in the TME through STAT3 activation Inhibition of T-cell function
Intracellular activation of *EGFR* signaling	↑ JAK/STAT3 ↑ PI3K/AKT/mTOR ↑ Ras/RAF/MEK/ERK ↑ NF-kB	↓ Down-regulation of MHC I/II class - ↓ Inhibition of STAT1 activity↑ MDSCs - ↓ DCs and APCs - ↑ TAM-M2 polarization↑/↓ PD-L1; ↓ IFNγ signature↑ Production and release of negative modulators (TGF-β, IDO, CCL-2)
Tumor mutational load and Neoantigens	↓ TMB	Low TMB may negatively influence the immune-mediated anti-tumor response
Dysregulation of the immunometabolism	↑ CD39/CD73 – adenosine signaling ↑ IDO ↑ ARG-1	↑ Immunosuppressive TME restraining anti-tumor immunity through A2AR↑ Degradation of tryptophan into immunosuppressive kynurenines↑ TAM-M2 polarization

*TME, tumor microenvironment; mAb, monoclonal antibody; EGFR, epidermal growth factor receptor; NSCLC, Non-Small-Cell Lung Cancer; MHC, major histocompatibility complex; CTLA4, cytotoxic T-lymphocyte antigen 4; PD-1, programmed death 1; LAG3, lymphocyte activation gene-3; TIM3, T cell immunoglobulin and mucin domain 3; VISTA, V-domain immunoglobulin suppressor of T-cell activation; PDL1, programmed death ligand 1; PDL2, programmed death ligand 2; IFN, interferon; MDSCs, myeloid-derived suppressor cells; Tregs, regulatory T cells; TAM, tumor-associated macrophages; MMP, matrix metalloproteinase; CCL-2, CC chemokine ligand-2; TGF, tumor necrosis factor; VEGF, vascular endothelial growth factor; iNOS, inducible nitric oxide synthase; IDO, indoleamine 2,3 dioxygenase; IL, interleukin; TMB, tumor mutation burden; A2AR, adenosine 2A receptor; ARG, arginase*.

In NSCLC, several studies reported that highly infiltrating T-lymphocytes in TME are related to the efficacy of immunotherapy and thus good prognosis (49). Indeed, several studies found significantly reduced CD8^+^ TILs in an *EGFR*-mutated NSCLC group compared with a wild-type *EGFR* group (17). In terms of *EGFR* mutation sites different immunological profiles have been reported with the prevalence of “inflamed” TME consisting of higher level of functional TILs in *EGFR*^*L*858*R*^ samples compared to *EGFR* exon 19 deletion tumor samples (50).

To support the evidence on the heterogeneity of *EGFR* mutations, Hastings et al. retrospectively analyzed clinical and molecular data on 171 cases of *EGFR*-mutant lung tumors treated with ICIs. Although *EGFR*-mutant tumors typically showed a low response to immunotherapy, clinical outcomes appear to vary by allele. In specific, *EGFR*^*L*858*R*^ tumors had a similar response rate and OS to an *EGFR* wild-type NSCLC population, while tumors harboring deletions in exon 19 cases did substantially worse (13).

Furthermore, it is noteworthy that the relationship between *EGFR* mutations and PD-L1 expression remains largely controversial, since pre-clinical data reported that the activation of *EGFR* signaling directly drives “intrinsic” PD-L1 up-regulation in a NSCLC model through several pathways such as PI3K/AKT/mTOR, Ras/RAF/MEK/ERK, JAK/STAT, and NF-kB (39). In addition, the activation of *EGFR* signaling may lead to the down-regulation of both class I and II antigens of the major histocompatibility complex (MHC) whose expression is regulated by the MEK/ERK pathway (51). Conversely, a large number of clinical trials reported that PD-L1 expression in *EGFR* wild-type tumors was significantly higher than in *EGFR* mutant NSCLC (52).

Regarding the field of predictive biomarkers research in NSCLC and other cancer types, tumor mutation burden (TMB) also emerged as a promising predictive biomarker of ICIs efficacy (53). TMB is the total number of insertion, deletion, and substitution mutations per megabase of the coding region of a tumor genome. Among patients with NSCLC treated with anti-PD-1 mAb, higher non-synonymous TMB seemed to be related with greater benefit, but a large phase III randomized trial did not confirm these preliminary observations (54). However, *EGFR*-addicted NSCLC were shown to have also low TMB (55).

Further, the activation of the *EGFR* oncogene pathway induces the release of several immunosuppressive factors to accomplish evasion of the host anti-cancer immune response. On the other hand, the inefficient killing of tumor cells is mostly due to a direct effect of *EGFR*-mutated lung cells through the over-production of negative modulators of immune cells including tumor necrosis factor-β (TGF-β), interleukin-10 (IL-10), vascular endothelial growth factor (VEGF), indolamine 2,3-dioxygenase (IDO), C-C chemokine ligand 2 (CCL-2), arginase (ARG)-1, and adenosine. The abundant immunosuppressive environment promotes both conversion and proliferation of CD3^+^CD4^+^CD25^−^ cells into CD4^+^CD25^+^FoxP3^+^ Tregs population, leading to immune suppression mediated by tumor release of *EGFR*-containing exosomes (47). Similarly, the increased bioavailability of soluble factors, such as IL-10, TGF-β, and CCL2, induces both recruitment and accumulation of immunosuppressive cell populations, such as MDSCs, into the TME, resulting in the activation of the signal transducer and transcriptional activator-3 (STAT3) pathway. Furthermore, STAT3 induces the activation of MDSCs, exerting additional immunosuppressive functions, such as inducing impairment of the antigen processing machinery mediated by dendritic cells (DCs), compromising T-cell-mediated cytotoxicity, inducing angiogenesis process by VEGF and matrix metalloproteinase (MMP) release, and promoting macrophage phenotype polarization in TAMs. Thus, this TME composition strongly restrains T-cell effector functions through paracrine signals that promote cancer growth (40).

Finally, in addition to the avoiding immune destruction (56, 57), a major emerging “hallmark” of immune evasion in TME harboring *EGRF* mutations is the reprogramming of immunometabolism (58) through CD39/CD73 ectonucleotidases complex that quickly converts adenosine triphosphate (ATP) in adenosine (59), one of most powerful known immunosuppressive metabolite (18, 60). Therefore, it is likely that this impairment in the immune system expressed as “uninflamed” TME and characterized by immunological tolerance provokes a weak immunogenicity leading to a poor responsiveness to immunotherapy in patients with *EGFR*-mutant NSCLC.

## Immunometabolism Mediated by CD39/CD73 - Adenosine Axis in *EGFR*-Mutant NSCLC

The hallmarks of cancer, defined as acquired functional capabilities allowing cancer cells to survive, proliferate, and spread, have been initially identified as modification in cells phenotype enabling replicative immortality, sustained proliferative signaling, evasion from growth suppressors, resistance to cell death, angiogenesis, invasion, and metastasis (56). In the last few decades, further hallmarks have been added to this list, including tumor-promoting inflammation, genome instability and mutation, evasion from immune destruction, and reprogramming of energy metabolism (57). During the multi-step tumorigenesis process, the disruption of energy metabolism is proving to be a cardinal feature in lung cancer development and is characterized by the engagement of the aerobic glycolysis, a process where the conversion of glucose into lactate occurs even in presence of sufficient oxygen to support glucose catabolism (Warburg effect) (61).

The TME could alter the immunometabolism and provide immunosuppressive metabolic substrates, thereby modifying the function of immune cells. On the other hands, T-cell subsets need alternative energetic pathways to satisfy their immune response efficiency and to balance the immune system activity. In this context, adenosine signaling involving CD39/CD73 ectonucleotidases expressed on various tumor cells is a critical pathway in TME to evade the immune surveillance and generate an immunosuppressive milieu (18). Specifically, in addition to the pleiotropic effects on immune cells infiltrating tumor, hypoxia directly induces increased adenine nucleotide in TME. Adenosine is an intra- and extra-cellular nucleoside that shows several effects in different tissues depending on its interaction with the following four G-protein-coupled adenosine receptors: A1, A2A, A2B, and A3 (62). In specific, ATP is degraded to adenosine by the ectonucleotidases complexes such as CD39 (ectonucleoside triphosphate diphosphohydrolase-1, E-NTPDase1) and CD73 (ecto-5′-nucleotidase, Ecto5′NTase), which convert ATP to adenosine monophosphate (AMP) and AMP to adenosine and inorganic phosphate, respectively. Adenosine is irreversibly deamidated to inosine by the adenosine deaminase (ADA) enzyme, while extra-cellular adenosine binds to adenosine receptors differently expressed by stromal and immune cells surrounding tumor, thus contributing to immune cell dysfunction (60). Interestingly, A2AR (A2A adenosine receptor) expression has also been found in human lung cancer cells, mostly in adenocarcinoma, exerting a partially direct effect on tumor growth while its antagonism induces tumor growth inhibition through apoptosis activation. Moreover, A2AR is the high-affinity adenosine receptor and appears to have the highest prevalence across all immune cells within both the adaptive and innate systems.

CD73 is endogenously expressed on endothelial cells, epithelial cells, and some immune subsets, and its expression has also been observed in several cancer types, including melanoma, colon, breast, ovarian, and lung cancer (19). Notably, the main drivers of CD73 expression within TME include hypoxia, TGF-β, type I IFN, IL-1, and prostaglandin (63).

Contrarily, so far little is known about the expression levels of CD39 on intra-tumoral T-cells in NSCLC. Recently, an interesting study reported a consistent co-expression of CD39 and PD-1 receptor on tumor-infiltrating immune cells in NSCLC TME than immune cells from normal lung tissue. CD39 was found also upregulated on several immune cells, including CD4^+^ and CD8^+^ T-cells, CD16^+^NK cells, macrophages, and B cells. Furthermore, CD39^+^FoxP3^+^ Tregs were highly enriched in the TME. Therefore, the CD39 upregulation on immune cells in TME suggests that the CD39 pathway may, in addition to PD-1 signaling, represent another relevant mechanism for tumor-induced immunosuppression in NSCLC (64). It is also demonstrated that tumor TGF-β induces CD39/CD73 over-expression on MDSCs in NSCLC TME via phosphorylation of mTOR, and subsequently activation of hypoxia-inducible factor-1 (HIF-1) signaling. Thus, TGF-β stimulating CD39/CD73 expression suppress both T-cells and NKs immunity (65). In addition, the increased expression of CD39 on cytotoxic T-cell induced by MDSCs correlated with poor prognosis in advanced NSCLC patients treated with anti-PD-1 mAbs (66).

Therefore, CD39/CD73 nucleotidases are not only involved in both purine and pyrimidine nucleotide synthesis but represents also a negative modulator of immune signaling through adenosine production in the TME (59). Accordingly, in TME the increased adenosine production mainly through A2AR activation on immune system cells impairs T-cell cytotoxicity, cytokine production, and T and NK cell function as well as induces suppression of antigen-presenting cells (APCs) (67). Indeed, in the myeloid compartment, adenosine skews the differentiation of DCs into tolerogenic and immunosuppressive DCs and enhances the immunosuppressive activity of TAM through macrophage M2 polarization. Adenosine release in TME also promotes Tregs and MDSCs proliferation (68) (see Figure 1). Moreover, recent studies have demonstrated that immunosuppressive populations including Tregs and MDSCs enhance their intrinsic suppressive activity also by direct CD73/CD39 upregulating on their cell surface. CD73 expression on FoxP3^+^ Tregs mediates part of their pro-tumorigenic effect converting proinflammatory extracellular ATP into powerful immunosuppressive adenosine (69).

In addition to traditional pathways mediated by CD39/CD73, an alternative enzymatic cascade has been recently reported and includes ectoenzymes such as CD38 (NAD^+^ nucleosidase), CD203α (ecto-nucleotide pyro-phosphatase phosphodiesterase 1) and CD73 (70, 71), thus participating to the composition of the so-called “purinergic milieu” (72). Indeed, in the search of a better understanding of the mechanisms involved in the acquired immunotherapy resistance, it has been reported that the unresponsiveness to PD-1/PD-L1 inhibition in lung murine models is probably mediated by the tumor up-regulation of CD38 enzyme, which is induced by all-trans retinoic acid and IFN-β in the TME. Therefore, CD38 represents an alternative ectoenzymatic pathway that limits the cytotoxic activity of CD8^+^ T-cells through the activation of adenosine receptor signaling (73), suggesting a potential role of CD38 blockade to overcome immune resistance (74).

Regarding the clinical significance of adenosine signaling, tumor CD73 expression has been widely associated with poor prognosis in several types of cancer including melanoma, colorectal, and triple-negative breast cancers (75). Indeed, to better define the relevance of adenosine signaling in lung cancer, an interesting relationship between immunohistochemistry (IHC) expression of CD73 in tumor tissues and clinical outcome has been found in patients with advanced NSCLC (stage I-III) (76). Moreover, high CD73 expression was an independent factor of poor prognosis in terms of OS and recurrence-free survival, thus exhibiting a remarkably worse prognostic meaning (76).

It is also noteworthy the relationship reported by Streicher et al. among the *EGFR* oncogene activation, the expression of CD73 and the reduced release of IFN-γ in NSCLC cell lines compared to wild-type cells. This exploratory analysis was conducted on tumor biopsies of advanced NSCLC patients from a non-randomized phase Ib/II clinical trial (NCT01693562) and from TGCA. According to these evidence, EGFR-mutant adenocarcinomas displayed >2-fold increased expression of CD73 compared to wild type, and this mechanism might, at least in part, explain their poor responsiveness to immunotherapy (19). Besides CD73 expression was induced by epidermal growth factor (EGF), and the pharmacological inhibition through *EGFR*-TKi induced its decrease in *EGFR*-mutated cancer cell lines.

A further study showed for the first time an inverse association between CD73 expression and activated tumor-infiltrating lymphocytes; in over 1,000 human lung cancer samples (77). It was demonstrated that high levels of CD73 significantly correlated with lower infiltration of activated CD8^+^ T-cells compared to those tumor samples with low CD73 expression. Interestingly, CD73 expression was significantly increased in samples with *EGFR* mutations when compared with wild type tumors (77). To confirm this evidence, it has been recently reported that lower levels of baseline tumor adenosine are associated with a reduced efficacy of anti- PD-1/CTLA-4 mAbs in cohorts of ICI-treated patients (HR = 0.29, *P* = 0.00012) (78).

By contrast, Ishii et al. retrospectively showed that high CD73 expression correlated with favorable clinical efficacy of immunotherapy in patients with *EGFR*-mutated NSCLC who have developed resistance to *EGFR*-TKIs, although this study had some limitations such as the limited number of patients analyzed (63).

## Targeting Immunosuppressive CD73 - Adenosine Axis in Lung Cancer: A New Strategy

The emerging role of CD39/CD73 - adenosine axis in *EGFR*-mutated NSCLC growth, progression, and ICI resistance has allowed to define a further immune checkpoint as a potential strategy to develop targeted treatments (79, 80).

Indeed, regarding the effect of adenosine signaling in the defective regulation of anti-tumor response observed in preclinical tumor models (81–83), different strategies targeting CD73 ectonucleotidase are currently under extensive clinical investigation also in advanced NSCLC (46). Given the broad expression of ectonucleotidases and adenosine receptors in the lung TME, a better understanding of their specific functions will be crucial to implement this new generation of immunological therapeutics (84).

In specific, the adenosine signaling inhibition is based on the use of either small molecule inhibitors or humanized mAbs in order to inhibit adenosine production in the TME or counteract adenosine pathway through targeting the adenosine receptors (85). Small molecules overcame mAbs in terms of both feasibility of administration and bioavailability. By contrast, anti-CD73 mAbs represent a valid alternative for their longer half-life and high specificity. In addition, both direct and indirect effects of mAbs on immune system cells as well as on target cells have been clearly demonstrated (86, 87).

Recent studies showed that the single A2AR blockade or the combination with either PD-1/PD-L1 or CTLA-4 mAbs induces T-cell proliferation, enhances the expression of IFNγ and granzyme B by tumor-infiltrating CD8^+^ T-cells, thus restraining the tumor growth in preclinical models (82, 83). Therefore, the therapeutic effect of A2AR antagonists may be maximized in “inflamed” tumors characterized by infiltrating tumor-reactive T-cells that are otherwise rendered impotent by high adenosine levels in the TME. Interestingly, the A2AR antagonism could prevent negative signaling in T-cells and inhibit angiogenesis process, but also play a direct inhibitory effect on lung cancer cells themselves (88). Anyway, the safety and efficacy of several A2AR inhibitors await evaluation in many ongoing trials also in advanced NSCLC treatment, as summarized in Table 3. Preliminary data demonstrated that A2AR inhibitor (CPI-444), as a single agent and in combination with the anti-PD-L1 mAb (Atezolizumab®) is well-tolerated and shows anti-tumor activity in refractory renal cancer and NSCLC cohorts (89).

**Table 3:** Current development status of Adenosine Receptor Antagonists in advanced non-small cell lung cancer.

**Clinical trial**	**Drug**	**Mechanism of action**	**Pharmaceutical company**	**Phase of development**	**Condition or disease**	**Drugs combination**
NCT02503774	AZD4635	A2AR antagonist	MedImmune	I/II	*EGFR*m NSCLC	AZD4635; MEDI9447 (Oleclumab); Osimertinib
NCT02403193	PBF-509	A2AR antagonist	Palobiofarma SL/Novartis	I/II	Advanced NSCLC *EGFR*^L858R/Del19mutations^	PBF-509; PDR001
NCT03454451	CPI-444	A2AR antagonist	Corvus pharmaceuticals	I	Advanced solid tumors	CPI-444; CPI-006; Pembrolizumab
NCT03549000	NIR178	A2AR antagonist	Surface oncology, novartis	I	NSCLC; TNBC; PDAC; Colorectal Cancer MSS; Ovarian Cancer; RCC; mCRPC	NIR178; NZV930; Spartalizumab
NCT03274479	PBF-1129	A2AR antagonist	Palobiofarma SL	I	Locally advanced or metastatic NSCLC	PBF-1129
NCT02655822	CPI-444	A2AR antagonist	Corvus pharmaceuticals	I/Ib	Advanced cancers	CPI-444; anti-PD-1/PD-L1 mAb

*Source from ClinicalTrials.gov*.

*mAb, monoclonal antibody; EGFR, epidermal growth factor receptor; NSCLC, Non-Small-Cell Lung Cancer; PDR001, anti-PD-1 mAb; CPI-006, anti-CD73 Fully mAb; TNBC, Triple Negative Breast Cancer; PDAC, Pancreatic Ductal Adenocarcinoma; MSS, Microsatellite Stable; RCC, Renal Cell Carcinoma; mCRPC, Metastatic Castration Resistant Prostate Cancer; NZV930, anti-CD73 Fully mAb*.

Finally, the current development of anti-CD73-based strategies and relative ongoing clinical trials in monotherapy or combination with immune checkpoint inhibitors as anti-PD-(L)1 mAbs or targeted therapies (*EGFR*-TKIs) in the treatment of advanced NSCLC are summarized in Table 4. The clinical results obtained from these trials might help to clarify the clinical relevance of CD73 as immune target in the treatment of patients with *EGFR*-mutated NSCLC.

**Table 4:** Current development status of anti-CD73 strategies in advanced non-small cell lung cancer.

**Clinical trial**	**Drug**	**Mechanism of action**	**Pharmaceutical company**	**Phase of development**	**Condition or disease**	**Drugs combination**
NCT02503774 NCT03736473 NCT03381274	MEDI9447 (Oleclumab)	Fully mAb	MedImmune	I/II I I	*EGFR*m NSCLC Advanced solid tumors Advanced solid tumors	MEDI9447; Osimertinib; AZD4635 MEDI9447; MEDI4736 MEDI9447
NCT02754141	BMS-986179	Fully mAb	Bristol-Myers Squibb	I/II	Advanced Solid Tumors	BMS-986179; Nivolumab
NCT03454451	CPI-006	Fully mAb	Corvus Pharmaceuticals	I	Advanced Solid Tumors	CPI-006; CPI-444; Pembrolizumab
NCT03549000	NZV930	Fully mAb	Surface Oncology, Novartis	I	NSCLC; TNBC; PDAC; Colorectal Cancer MSS; Ovarian Cancer; RCC; mCRPC	NZV930; NIR178; Spartalizumab
NCT04148937	LY3475070	CD73 inhibitor	Eli Lilly and Company	I	Advanced solid tumors	LY3475070; Pembrolizumab
NCT03835949	TJ004309 (TJD5)	Fully mAb	I-Mab Biopharma, TRACON Pharmaceuticals	I	Advanced solid tumors	TJD5; Atezolizumab
_	IPH5301	Fully mAb	Innate Pharma	Pre-clinical	_	_
_	AB680	CD73 inhibitor	Arcus	Pre-clinical	_	_

*Source from ClinicalTrials.gov*.

*mAb, monoclonal antibody; EGFR, epidermal growth factor receptor; NSCLC, Non-Small-Cell Lung Cancer; AZD4635, oral adenosine A2A receptor antagonist; MEDI4736, Durvalumab; CPI-444, Adenosine A2A receptor antagonist; TNBC, Triple Negative Breast Cancer; PDAC, Pancreatic Ductal Adenocarcinoma; MSS, Microsatellite Stable; RCC, Renal Cell Carcinoma; mCRPC, Metastatic Castration Resistant Prostate Cancer; NIR178, oral adenosine A2A receptor antagonist*.

To date, preliminary results suggest that anti-CD73 mAbs and PD-1 blockade represent a promising approach with an acceptable safety profile in several tumor subtypes such as metastatic melanoma (90), encouraging future therapeutic applications in the clinical practice.

## Conclusions

Lung tumor remains the most aggressive and life-threatening cancer, although significant therapeutic improvements have been obtained in the last years. To this regard, immunotherapy combinations are promising strategies aimed at restoring the anti-cancer immunity as well as overcoming innate and adaptive resistance to ICIs. In the era of successful immunotherapy, while non-oncogene addicted advanced NSCLC obtains a great survival benefit, the effectiveness of immunotherapy in *EGFR*-mutated NSCLC appears disappointing. Indeed, the anti-PD-(L)1 monotherapy has been shown to have minimal activity in *EGFR*-mutant NSCLC and therefore should only be considered after all other therapies that have been shown to be more effective in this patient population, such as *EGFR*-TKIs, platinum-doublet chemotherapy, and probably docetaxel plus anti-angiogenic drug (Ramucirumab®), have been exhausted.

The identification of additional biomarkers that are predictive of benefit to anti-PD-1 mAbs would be an important advance in our understanding, especially considering the potential lethal immune-toxicities and high cost of ICIs.

Therefore, a better understanding of biological mechanisms involved in tumor progression and immune evasion as well as of the dynamic hallmarks of *EGFR*-mutated NSCLC TME is required. The “uninflamed” TME and the low TMB are typical features of *EGFR*-mutated NSCLC, potentially explaining the impaired response to immunotherapies.

To this regard, an interesting hallmark of NSCLC-harboring oncogenic driver mutations, leading to immune escape, and conferring primary immune resistance, is the reprogramming of energy metabolism mediated by CD39/CD73—adenosine signaling. In order to reinforce the critical role of adenosine signaling in tumorigenesis, several trials have demonstrated that CD73 over-expression by cancer cells correlated with poor prognosis in NSCLC patients. Furthermore, in advanced NSCLC recent findings identified a novel relationship between *EGFR* oncogene activation, over-expression of immunosuppressive molecule such as CD73 and reduced expression of IFNγ signature, and this may explain, at least in part, the limited responsiveness to immunotherapy in *EGFR*-mutated NSCLC. Despite these preliminary evidence, the exact relevance of CD73-adenosine signaling in both *EGFR*-mutated tumor cells and infiltrating immune cells to the efficacy of immune checkpoint inhibitors remains unclear. Anyway, in the preclinical studies the pharmacological antagonism of CD39/CD73—adenosine signaling potentiated anti-tumor responses in preclinical models that otherwise failed to respond to anti-PD-1/PD-L1 inhibition. These data agree with observations that blockade of other immune checkpoint inhibitors over-expressed in the TME, such as CTLA-4, TIM-3, TIGIT can enhancing IFNγ production by CD8^+^ TILs following anti-PD-1 inhibition.

In conclusion, adenosinergic signaling is emerging as a powerful immune-metabolic checkpoint in advanced NSCLC. Hence, further pre-clinical data and clinical trials aiming at translating adenosine signaling inhibition strategies in *EGFR*-mutated NSCLC are needed to target the dysregulated immunometabolism in the TME, in order to overcome primary resistance to immunotherapy.

## Author Contributions

All authors listed have made a substantial, direct and intellectual contribution to the work, and approved it for publication.

## Conflict of Interest

CG received honoraria as speaker bureau and advisory board member from Astra Zeneca, BMS, MSD, and Roche. The remaining authors declare that the research was conducted in the absence of any commercial or financial relationships that could be construed as a potential conflict of interest.
